# Phylogenetic analysis based on whole genome sequence of bovine leukemia virus in cattle under 3 years old with enzootic bovine leukosis

**DOI:** 10.1371/journal.pone.0279756

**Published:** 2023-01-25

**Authors:** Masaki Maezawa, Yuki Fujii, Masataka Akagami, Junko Kawakami, Hisashi Inokuma

**Affiliations:** 1 Laboratory of OSG Veterinary Science for Global Disease Management, Graduate School of Agricultural and Life Sciences, The University of Tokyo, Bunkyo-ku, Tokyo, Japan; 2 Ibaraki Prefecture Kenpoku Livestock Hygiene Service Center, Mito, Ibaraki, Japan; 3 Laboratory of Farm Animal Medicine, Graduate School of Agricultural and Life Sciences, The University of Tokyo, Bunkyo-ku, Tokyo, Japan; Shanxi University, CHINA

## Abstract

Enzootic bovine leukosis (EBL) is one of bovine neoplasms caused by bovine leukemia virus (BLV). Although EBL is typically observed in cattle over 3 years old, several cases of EBL onset in cattle under 3 years old have been reported in Japan. The mechanism for EBL onset in young cattle remains unclear. Although genetic variation of BLV is limited, the variations could affect viral properties relating to BLV pathogenesis. The purpose of this study was to clarify relationship between early onset of EBL and BLV groups. Moreover, we also aimed to characterize BLV that cause early onset of EBL. Whole genome sequences of BLV in 72 EBL cattle under 3 years old and 50 EBL cattle over 3 years old were identified. Phylogenetic analysis showed that BLV was divided into 4 groups (A, B-1, B-2 and Other). The BLV from EBL cattle under 3 years old were mainly classified as group A and B-1, while those from EBL cattle over 3 years old were mainly included in group B-2. Common sequence of group A and B-1 was compared with those of group B-2. Specific sequences in LTRs, *gag-pro-pol*, env and *tax* gene regions were identified in these groups. Amino acid substitutions of Pro and Tax protein were predicted in those nucleotide sequences. Those genetic variations might contribute to the early onset of EBL.

## Introduction

Bovine leukemia virus (BLV) belongs to the family Retroviridae genus Deltaretrovirus and is the causative agent of enzootic bovine leukosis (EBL) [1]. Although almost all BLV-infected animals remain clinically asymptomatic throughout their lifespan, less than 5% of the infected cattle develop EBL [2]. Due to the lengthy latency period required by BLV, EBL is commonly seen in cattle older than 3 years [3], while onset of EBL in cattle under 3 years old has also been observed in Japan [4–6]. Although some factors related to onset of EBL in young cattle have been reported [6–8], the mechanism of early EBL onset is not fully understood.

BLV has been previously classified into three groups (A, B, and C) based on the complete genome sequence, even though genetic variation in BLV is limited [9, 10]. Moreover, viral replication activity in groups A, B, and C were high, moderate, and low, respectively [10]. Because proviral and disease progression were positively correlated, the pathogenicity might be different among groups. In addition, a case of EBL caused by BLV classified as group A in young cattle has been reported [6]. However, the relationship between early onset of EBL and BLV groups has been unclear. In the present study, we identified whole genome sequences of BLV in EBL cattle under and over 3 years of age and performed phylogenetic analysis to clarify the relationship between BLV groups and the age of EBL onset.

## Materials and methods

### EBL samples

Several organs from 122 cattle with suspicions of bovine lymphoma were provided by meat hygiene inspection centers and Livestock Hygiene Service Centers in Ibaraki, Iwate and Hokkaido, Japan. Samples included lymph nodes (mediastinal, superficial, subiliac, medial, and iliac lymph nodes), spleen, and solid tumors in several organs (heart, lung, abomasum, liver, kidney, and uterus) in 72 cattle under 3 years old and 50 cattle over 3 years old (S1 and S2 Tables). Pathological findings and/or B-cell clonality test confirmed the presence of B-cell lymphoma in all cattle [11]. BLV copy numbers in all cattle were over 2,000 per 50 ng DNA used as the diagnostic criterion for BLV associated with tumor development in a previous study [4]. Genomic DNA was extracted from the tissues using QIAamp DNA Mini Kit (QIAGEN, GmbH, Hilden, Germany) and stored at -30°C.

### Sequencing of the BLV proviral genome

The genomic DNA was used as a template for the PCR, which was performed using PrimeSTAR^®^ GXL DNA polymerase (Takara Bio, Shiga, Japan) and two primer pairs (BLV 1–17 F: 5′-TGTATGAAAGATCATGC-3′ and BLV 4565–4586 R: 5′-AATCTGATTGTGAGTCCAGAGG-3′, and BLV 4416–4436 F: 5′-CAGTTCGGAGTTTCCCTTTCT-3′ and BLV 8703–8720 R: 5′-TGTTTGCCGGTCTCTCCT-3′) [10]. The PCR reaction was as follows: amplification with 30 cycles of denaturation at 98°C for 10 sec, annealing at 55°C for 15 sec and extension at 68°C for 5 min, and final extension at 68°C for 2 min. PCR products were treated with ExoSAP-IT Express (Thermo Fisher Scientific, Waltham, MA, USA). DNA libraries were prepared using QIAGEN^®^ QIAseq FX DNA Library Kit (QIAGEN) following the manufacturer’s protocol and sequenced on an Illumina iSeq system using 2 × 150 bp paired-end reads. Quality control procedures were performed using the Trim Reads tool in the CLC Genomics Workbench v. 20.0 (CLC bio, Aarhus, Denmark) (CLC). Unless otherwise stated, all software was used with default values applied. Mapping of quality filtered reads against a reference BLV genome (Accession No. EF600696.1) was performed using the CLC mapping tool and the whole genome sequence of BLV in all samples was identified. Coverages of all samples were over 100. The whole BLV genome sequences of the 122 BLV identified in this study were deposited in the GenBank database under the accession numbers LC733242 to LC733363.

### Phylogenetic analysis

A phylogenetic tree was constructed by neighbor-joining methods (1,000 bootstrap replications) using the whole genome sequence of BLV in all samples and complete genome sequences of BLV obtained from GenBank database under accession numbers LC164086 and AP018006 to AP018032. Based on the previous study, BLV were classified into 4 groups (A, B, C and Other) [10].

### Alignment of amino acid and nucleotide sequences

Editing and alignment of nucleotide and amino acid sequences were performed using CLC and MEGA 7.0 software [12].

## Results

### Identification of nucleotide insertions and deletions in BLV genome sequences

**Fig 1:**
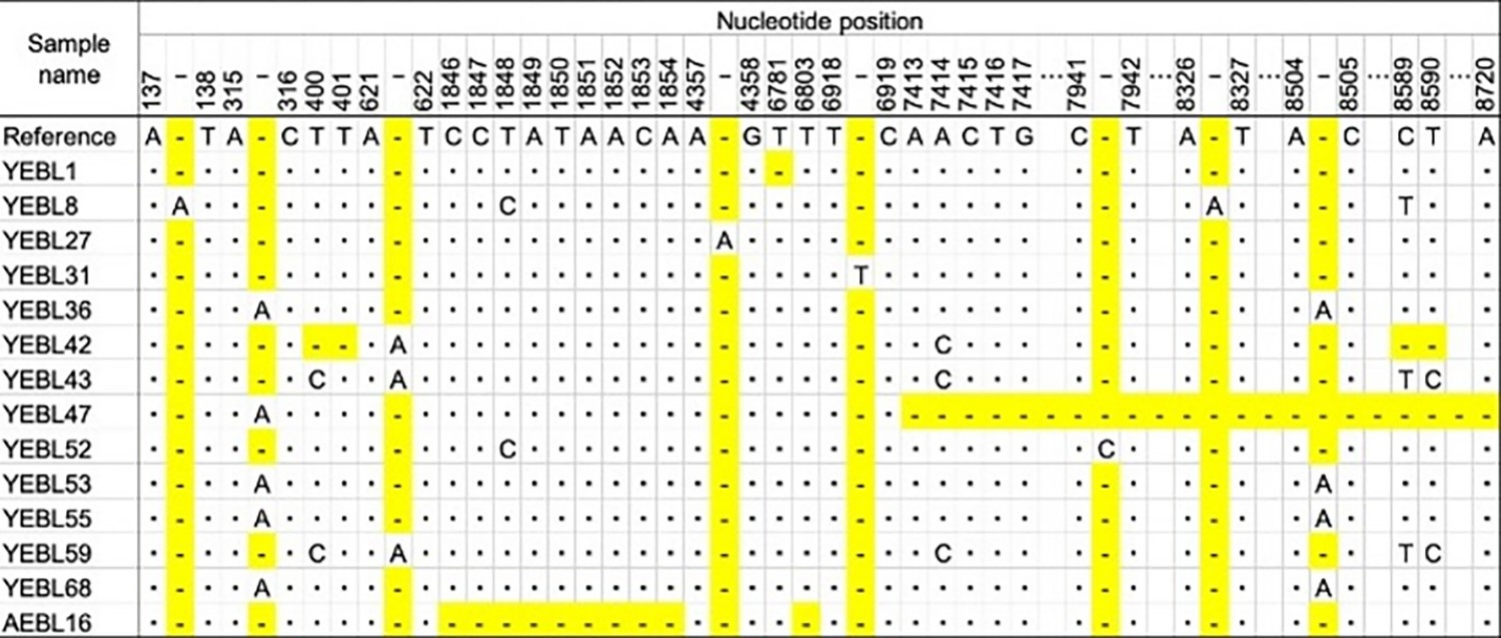
Summary of nucleotide insertions and deletions. The dots (”∙“) and the dash (”-“) indicate the same nucleotide and a nucleotide deletion, respectively.

The sequence of the integrated BLV provirus in 108 EBL cattle was 8720 bp in length and insertion and/or deletion were observed in 14 EBL cattle. Insertion of adenine at nucleotide position 137–138 in YEBL8, at 315–316 in YEBL36, 47, 53, 55 and 68, at 621–622 in YEBL42, 43 and 59, at 4357–4358 in YEBL27, at 8326–8327 in YEBL8 and at 8504–8505 in YEBL36, 53, 55 and 68, insertion of thymine at nucleotide position 6918–6919 in YEBL31 and insertion of cytosine at nucleotide position 7923–7924 in YEBL52 were observed (Fig 1). Deletion at nucleotide position 400–401 in YEBL42, at 1846–1854 in AEBL16, at 6781 in YEBL1, at 6803 in AEBL16, at 7413–8720 in YEBL47 and at 8589–8590 in YEBL42 were identified (Fig 1).

### The categorization of the BLV groups

**Fig 2:**
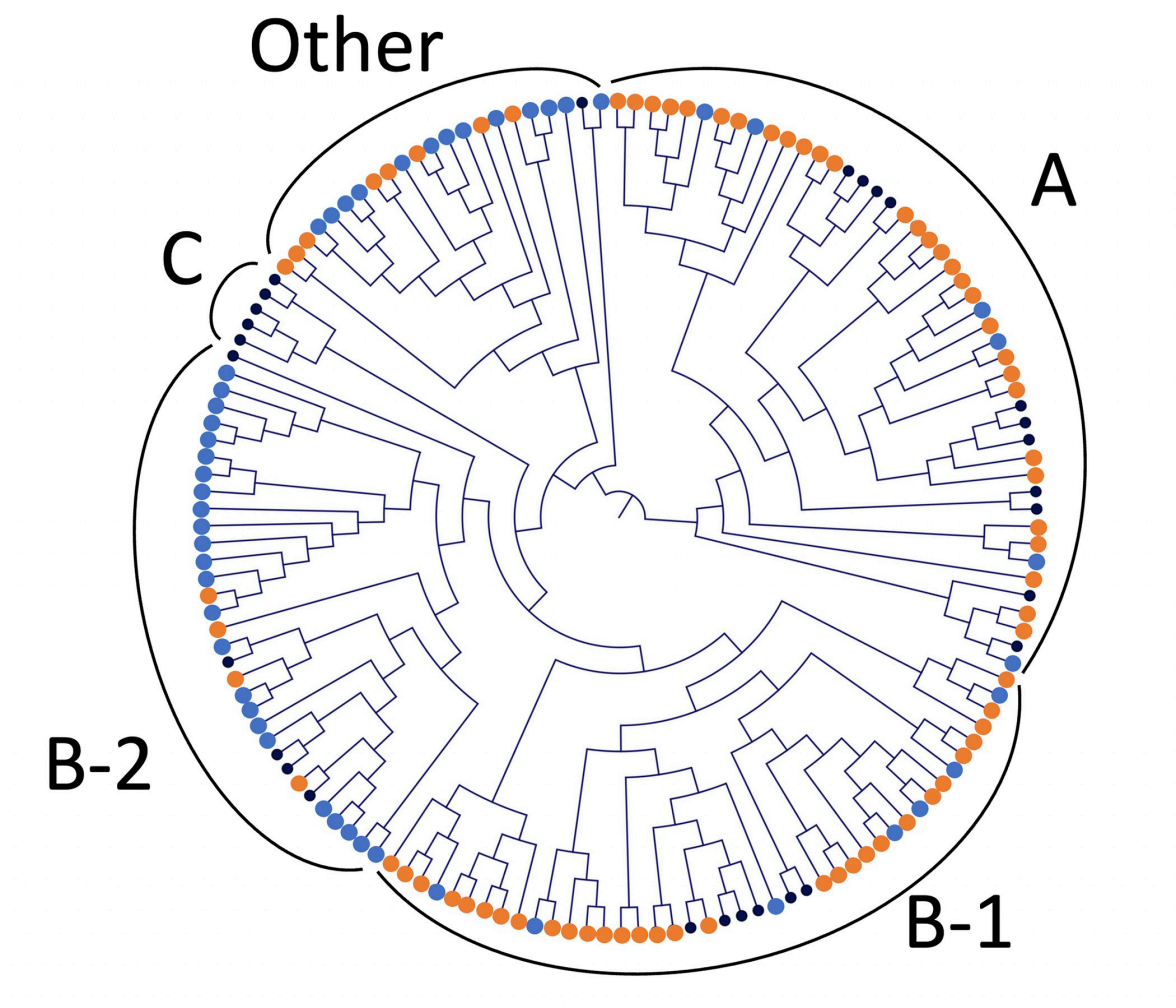
Phylogenetic analysis of the whole BLV genome sequences. A maximum-likelihood phylogenetic tree was constructed from whole BLV genome sequences of 72 EBL cattle under 3 years old, 50 EBL cattle over 3 years old and 28 reference sequences. The BLV were divided into 5 groups (A, B-1, B-2, C and Other). BLV from EBL cattle under 3 years old, EBL cattle over 3 years old and references were shown by red, blue and black circles, respectively.

Phylogenetic analysis showed that 36 and 65 of 122 BLV were classified into A and B group, respectively (Fig 2). Thirty BLV nucleotide sequences from cattle under 3 years old and 6 nucleotide sequences from cattle over 3 years old were classified as group A, and the group B were constructed 34 nucleotide sequences from cattle under 3 years old and 31 nucleotide sequences from cattle over 3 years old. Moreover, group B was divided into B-1 and B-2 groups. B-1 group was composed mainly of BLV from young EBL (30 nucleotide sequences from cattle under 3 years old and 7 nucleotide sequences from cattle over 3 years old) while B-2 group was composed mainly of BLV from old EBL cattle (4 nucleotide sequences from cattle under 3 years old and 24 nucleotide sequences from cattle over 3 years old). No BLV nucleotide sequences were classified as group C and the other 21 BLV nucleotide sequences did not belong to group A, B and C.

### Analysis of nucleotide and amino acid sequences

**Fig 3:**
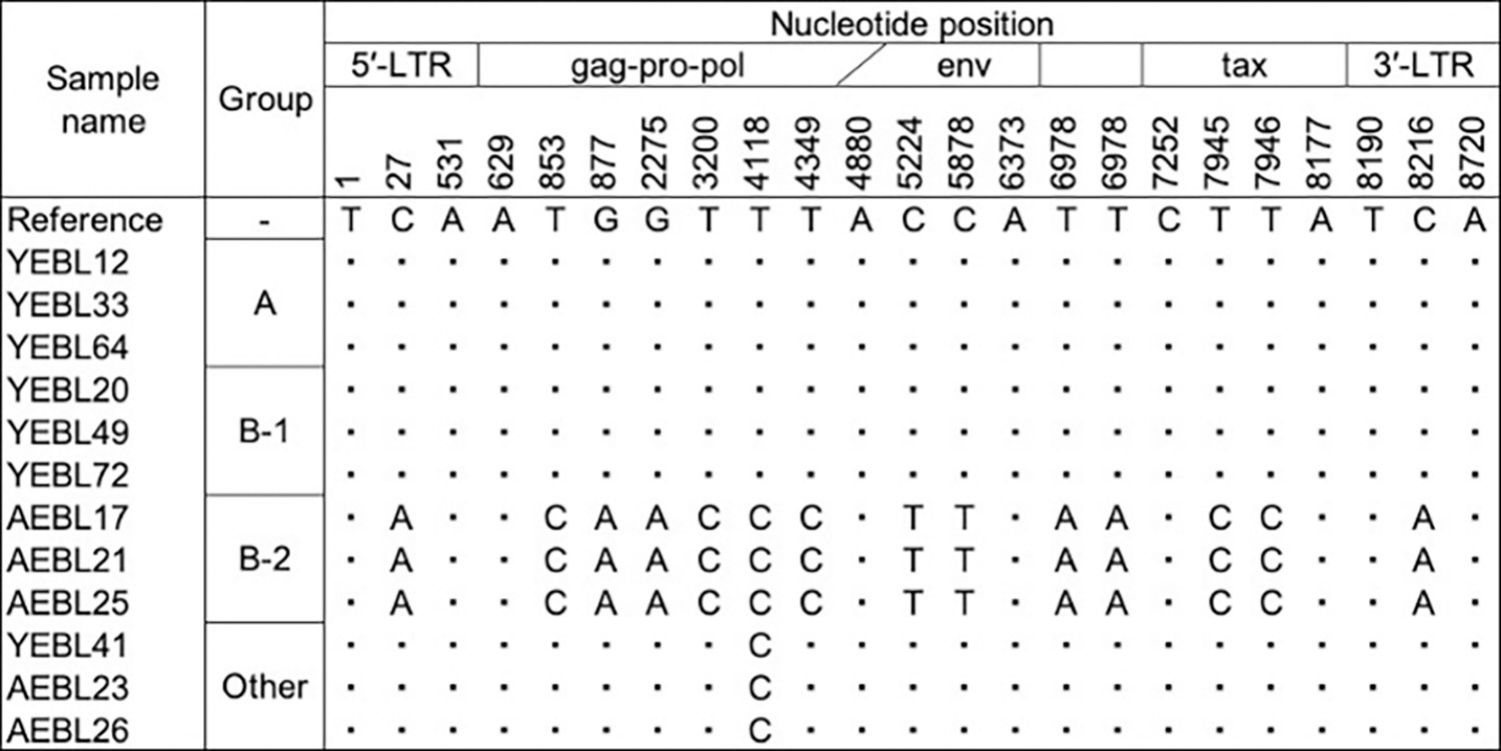
Summary of representative nucleotide substitutions among groups A, B-1, B-2 and Other. The dots (”∙“) indicate the same nucleotide.

To identify specific nucleotide substitutions between BLV genomes from EBL cattle under and over 3 years old, the common sequence of group A and B-1 was compared with those of group B-2. The specific nucleotide substitutions were identified in the nucleotide position at 27 (A and B-1: cytosine and B-2: adenine), 853 (A and B-1: thymine and B-2: cytosine), 877 (A and B-1: guanine and B-2: adenine), 2275 (A and B-1: guanine and B-2: adenine), 3200 (A and B-1: thymine and B-2: cytosine), 4118 (A and B-1: thymine and B-2: cytosine), 4349 (A and B-1: thymine and B-2: cytosine), 5224 (A and B-1: cytosine and B-2: thymine), 5878 (A and B-1: cytosine and B-2: thymine), 6978 (A and B-1: thymine and B-2: adenine), 7945 (A and B-1: thymine and B-2: cytosine), 7946 (A and B-1: thymine and B-2: cytosine) and 8216 (A and B-1: cytosine and B-2: adenine) (Fig 3). These nucleotide substitutions were located in 5′ and 3′ long terminal repeat (LTR), *gag-pro-pol*, *env* and *tax* regions. The substitutions of nucleotide position at 2275, 7945 and 7946 were predicted to cause amino acid substitution at the positions 158 of Pro (A and B-1: alanine and B-2: threonine) and 233 of Tax protein (A and B-1: leucine and B-2: proline) while the other nucleotide substitutions were not expected to affect amino acids.

## Discussion

BLV can be divided into three groups (A, B, and C) based on the whole genome sequence, and the difference of viral transmissibility among those groups has been reported [10]. However, there are few studies about pathogenicity among the group and the relationship between BLV groups and the age of EBL onset is unclear. In the present study, the BLV from EBL cattle under 3 years old were mainly classified as group A and B-1, while those from EBL cattle over 3 years old were mainly classified as group B-2. Therefore, the pathogenicity of BLV from group A and B-1 might be higher than those from group B-2. Moreover, no BLV from EBL cattle were classified as group C in the present study, and BLV in group C were isolated only from cattle without EBL in the previous study [10]. Those results indicated that the pathogenicity of BLV in group C might be low.

In the BLV genome, several functional genes and regions are contained [13, 14]. Both the 5′ and 3′ extremities of BLV genome present a sequence called LTR which includes transcriptional regulatory sequences and a point mutation in LTR region affects viral activity [15]. BLV has the *gag-pro-pol*, *env* and nonstructural genes from 5′ and 3′ of the genome. The *gag*, *pro*, *pol*, and *env* genes encode the internal structural proteins of the virion, the viral protease, the reverse transcriptase, and the envelope glycoproteins of the virion, respectively. The nonstructural genes *R3*, *G4*, *tax*, and *rex* are encoded within the *pX* region which is located between the *env* and 3′ LTR. Proteolytic processing at specific sites in the Gag protein and Gag-Pro-Pol precursors by the viral protease is an essential step in the viral life cycle [16]. The Tax protein functions as a transactivator of viral gene transcription and modulates the expression of several cellular genes. Therfore, the Tax protein is considered to play a crucial role in the leukemogenesis caused by deltaretroviruses [17–19]. The latency period of BLV encoding a proline at residue 233 of the Tax protein (P233-Tax) is approximately 2 years longer than that of BLV encoding L233-Tax [20]. In the present study, the substitutions of nucleotide sequence at LTRs, *pro* and *tax* were identified between common sequence of group A and B-1 and those of group B-2. Moreover, amino acid mutations of Pro and Tax protein were induced by nucleotide sequence substitutions. Those genetic variations might contribute to onset of EBL in young cattle.

Human T-cell leukemia virus type 1 (HTLV-1), closely related to BLV, has been implicated in adult T-cell lymphoma (ATL). Inactivation of the tax gene expression were observed by genetic and epigenetic alterations, including DNA methylation, histone modification, and deletion of the 5’-LTR in some ATL cells [21–25]. In more than half of ATL cases, two types of defective HTLV-1 proviral genomes were found [21]. The first defective type retained both LTRs and lacked internal sequences and the second defective type lacked 5′-LTR and internal sequences [21]. 5’-LTR is critical for transcription of viral genes, and the loss indicates that viral proteins, including Tax protein, cannot be transcribed from viral promoter in these cells. Even cells with the second defective type HTLV-1 provirus were found not to produce Tax protein in vitro [26]. The lack of Tax protein expression was also observed in majority of BLV-induced malignancies [27]. In the present study, defective BLV proviral genome including *tax* gene and 3’-LTR region were dentified in only YEBL47. Moreover, no 5’-LTR region defect was observed in all BLV. The different frequency and regions of defects between BLV and HTLV-1 suggested that both viruses might have different strategies for suppressing *tax* gene expression. Further studies are needed to clarify the mechanism to silence the *tax* gene transcription in BLV.

In conclusion, the present study demonstrated BLV with propensity for onset of EBL in cattle under 3 years old. Moreover, specific sequences in LTRs, *gag-pro-pol*, env and *tax* gene regions were identified in these BLV. Amino acid substitution of pro and tax were predicted in these nucleotide sequences. However, the influence of each of these genetic variants on pathogenicity of BLV was not evaluated in the present study. Further investigation is required to clarify the relationship between pathogenesis and these genetic variants.

## Supporting information

S1 Table BLV strains in EBL cattle under 3 years old. (PDF)

S2 Table BLV strains in EBL cattle over 3 years old. (PDF)
