# Supplementary material for: Phylogenetic analysis based on whole genome sequence of bovine leukemia virus in cattle under 3 years old with enzootic bovine leukosis
Source: PLoS One. 2023 Jan 25;18(1):e0279756. doi: 10.1371/journal.pone.0279756 (PMC9876212; doi:10.1371/journal.pone.0279756)
Supplement: S1 Table — (PDF) [file pone.0279756.s001.pdf]

Table S1. BLV strains in EBL cattle under 3 years old.

Cattle ID	Breed	Age (month)	BLV Group	Accession No
YEBL1	HF*	13	B-1	LC733242
YEBL2	JB*	21	A	LC733243
YEBL3	HF	22	Other	LC733244
YEBL4	JB	23	B-2	LC733245
YEBL5	HF	22	Other	LC733246
YEBL6	HF	14	Other	LC733247
YEBL7	F1*	17	B-1	LC733248
YEBL8	F1	25	A	LC733249
YEBL9	JB	30	A	LC733250
YEBL10	F1	10	B-1	LC733251
YEBL11	F1	18	B-1	LC733252
YEBL12	F1	8	A	LC733253
YEBL13	JB	21	A	LC733254
YEBL14	JB	27	A	LC733255
YEBL15	F1	25	A	LC733256
YEBL16	F1	23	B-1	LC733257
YEBL17	JB	23	B-2	LC733258
YEBL18	JB	23	A	LC733259
YEBL19	JB	28	A	LC733260
YEBL20	F1	17	B-1	LC733261
YEBL21	F1	23	B-1	LC733262
YEBL22	F1	33	B-1	LC733263
YEBL23	F1	34	B-1	LC733264
YEBL24	F1	22	B-1	LC733265
YEBL25	JB	23	A	LC733266
YEBL26	HF	18	A	LC733267
YEBL27	HF	31	Other	LC733268
YEBL28	HF	19	A	LC733269
YEBL29	JB	24	A	LC733270
YEBL30	HF	15	Other	LC733271
YEBL31	HF	13	Other	LC733272
YEBL32	F1	24	B-1	LC733273
YEBL33	F1	20	A	LC733274
YEBL34	HF	20	A	LC733275
YEBL35	F1	26	A	LC733276
YEBL36	HF	32	B-1	LC733277

YEBL37	JB	25	A	LC733278
YEBL38	F1	20	A	LC733279
YEBL39	HF	27	B-1	LC733280
YEBL40	JB	25	A	LC733281
YEBL41	JB	31	Other	LC733282
YEBL42	F1	21	B-1	LC733283
YEBL43	F1	22	B-1	LC733284
YEBL44	F1	23	A	LC733285
YEBL45	HF	19	A	LC733286
YEBL46	HF	19	B-1	LC733287
YEBL47	F1	21	B-1	LC733288
YEBL48	F1	27	A	LC733289
YEBL49	F1	27	B-1	LC733290
YEBL50	F1	27	B-1	LC733291
YEBL51	F1	21	B-1	LC733292
YEBL52	F1	21	A	LC733293
YEBL53	F1	35	B-1	LC733294
YEBL54	HF	19	A	LC733295
YEBL55	HF	20	B-1	LC733296
YEBL56	F1	27	A	LC733297
YEBL57	F1	24	A	LC733298
YEBL58	F1	27	B-1	LC733299
YEBL59	F1	30	B-1	LC733300
YEBL60	JB	28	B-1	LC733301
YEBL61	JB	27	B-1	LC733302
YEBL62	HF	19	Other	LC733303
YEBL63	HF	20	A	LC733304
YEBL64	JB	27	A	LC733305
YEBL65	HF	28	B-2	LC733306
YEBL66	HF	27	B-1	LC733307
YEBL67	JB	25	A	LC733308
YEBL68	JB	31	B-1	LC733309
YEBL69	HF	15	A	LC733310
YEBL70	HF	18	B-2	LC733311
YEBL71	HF	21	B-1	LC733312
YEBL72	HF	20	B-1	LC733313

* HF: Holstein-Frisian, JB: Japanese Black, F1: crossbreeds of HF and JB
