# Supplementary material for: Phylogenetic analysis based on whole genome sequence of bovine leukemia virus in cattle under 3 years old with enzootic bovine leukosis
Source: PLoS One. 2023 Jan 25;18(1):e0279756. doi: 10.1371/journal.pone.0279756 (PMC9876212; doi:10.1371/journal.pone.0279756)
Supplement: S2 Table — (PDF) [file pone.0279756.s002.pdf]

Table S2. BLV strains in EBL cattle over 3 years old.

Cattle ID	Breed	Age (month)	BLV Group	Accession No
AEBL1	HF*	38	B-1	LC733314
AEBL2	HF	46	B-2	LC733315
AEBL3	HF	46	A	LC733316
AEBL4	JB*	134	Other	LC733317
AEBL5	HF	101	A	LC733318
AEBL6	HF	47	B-1	LC733319
AEBL7	JB	108	B-2	LC733320
AEBL8	HF	81	B-1	LC733321
AEBL9	JB	48	B-2	LC733322
AEBL10	HF	41	Other	LC733323
AEBL11	JB	65	B-2	LC733324
AEBL12	JB	107	B-2	LC733325
AEBL13	HF	89	B-1	LC733326
AEBL14	HF	47	Other	LC733327
AEBL15	JB	140	B-2	LC733328
AEBL16	HF	55	A	LC733329
AEBL17	HF	62	B-2	LC733330
AEBL18	HF	37	Other	LC733331
AEBL19	JB	84	B-2	LC733332
AEBL20	JB	129	B-2	LC733333
AEBL21	JB	61	B-2	LC733334
AEBL22	HF	50	B-2	LC733335
AEBL23	JB	58	Other	LC733336
AEBL24	HF	94	Other	LC733337
AEBL25	JB	175	B-2	LC733338
AEBL26	JB	95	Other	LC733339
AEBL27	JB	107	B-2	LC733340
AEBL28	JB	49	B-2	LC733341
AEBL29	JB	117	B-2	LC733342
AEBL30	JB	40	B-2	LC733343
AEBL31	JB	95	B-2	LC733344
AEBL32	JB	83	B-2	LC733345
AEBL33	JB	180	B-2	LC733346
AEBL34	HF	42	Other	LC733347
AEBL35	HF	48	Other	LC733348
AEBL36	JB	154	B-2	LC733349

AEBL37	HF	82	Other	LC733350
AEBL38	JB	38	B-2	LC733351
AEBL39	JB	89	A	LC733352
AEBL40	JB	69	B-1	LC733353
AEBL41	HF	83	A	LC733354
AEBL42	JB	101	B-2	LC733355
AEBL43	HF	69	Other	LC733356
AEBL44	JB	109	B-2	LC733357
AEBL45	JB	90	B-2	LC733358
AEBL46	HF	48	Other	LC733359
AEBL47	HF	53	A	LC733360
AEBL48	HF	43	B-1	LC733361
AEBL49	JB	84	B-1	LC733362
AEBL50	HF	49	Other	LC733363

* HF: Holstein-Frisian, JB: Japanese Black
